# Utility of pH monitoring in surgical decision-making and outcome prediction in revisional antireflux surgery for anatomical failure

**DOI:** 10.1007/s00464-025-11857-4

**Published:** 2025-06-12

**Authors:** Inanc S. Sarici, Sven E. Eriksson, Naveed Chaudhry, Ping Zheng, Johnathan Nguyen, Shahin Ayazi

**Affiliations:** 1 Foregut Division, Surgical Institute, Allegheny Health Network, 4815 Liberty Avenue, Suite 439, Pittsburgh, PA 15224 USA; 2 Chevalier Jackson Esophageal Research Center, Western Pennsylvania Hospital, Pittsburgh, PA USA; 3 Department of Surgery, Drexel University, Philadelphia, PA USA

**Keywords:** Bravo pH monitoring, Antireflux surgery, Nissen fundoplication, Revisional surgery

## Abstract

**Background:**

Antireflux surgery (ARS) is effective for controlling GERD, but 10–20% of patients experience anatomical failure, and 3–7% eventually require revisional surgery. While pH monitoring is routinely used preoperatively, its role in guiding revisional ARS remains unclear. This study aimed to evaluate the role of pH monitoring in predicting the need for and outcomes of revisional ARS.

**Methods:**

We reviewed 278 patients (68% female, mean age 55) with anatomical failure after fundoplication who underwent 48-h pH monitoring from 2015 to 2023. Patients were stratified by DeMeester score at failure: normal vs. abnormal (≥ 14.7). Primary outcome was need for revisional ARS. Secondary outcome was favorable outcome at 1-year post-revision, defined as freedom from PPIs and patient satisfaction. Multivariable logistic regression evaluated the impact of pH monitoring on need for and outcome of revisional ARS.

**Results:**

Abnormal DeMeester scores were found in 132 patients (47.5%), who had higher rates of simultaneously herniated and disrupted fundoplication (48.5 vs. 24.0%, *p* < 0.001), longer median (IQR) time to failure [54.9 (20.9–121.0) vs. 27.9 (14.8–77.8) months, *p* = 0.004], and higher GERD-HRQL heartburn scores (*p* < 0.05). These patients were more likely to undergo revisional ARS (68.9 vs. 47.3%, *p* < 0.001), confirmed on multivariable analysis [OR 2.36 (1.28–4.37), *p* = 0.006].

At 14 (3) months post-revision, patients with abnormal DeMeester scores had higher rates of patient satisfaction (82.9 vs. 65.5%, *p* = 0.026) and freedom from PPIs (77.6 vs. 60.3%, *p* = 0.037) with lower GERD-HRQL total scores [7.0 (2.0–21.5) vs. 14.0 (6.0–32.0), *p* = 0.003]. Abnormal DeMeester score was the strongest predictor of favorable outcomes after revisional ARS [OR 3.98 (1.75–9.04), *p* = 0.001].

**Conclusion:**

Abnormal DeMeester score at time of failure predicts need for revisional ARS and is the strongest predictor of favorable outcome after revisional ARS, underscoring its role in surgical decision-making after failure.

**Graphical abstract:**

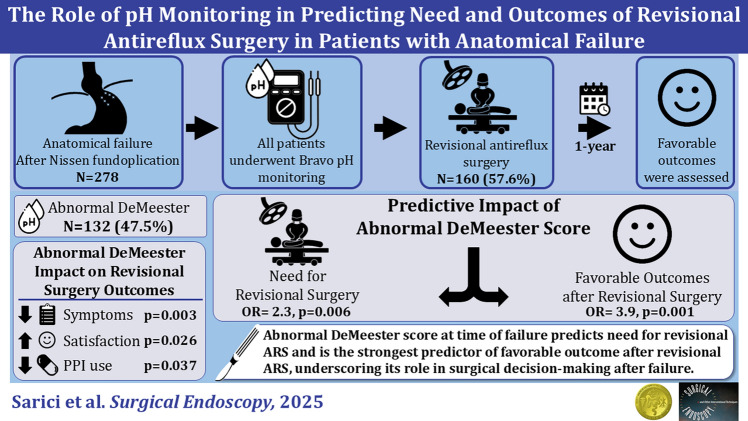

Laparoscopic Nissen fundoplication is the standard surgical approach for patients with gastroesophageal reflux disease (GERD). It is a safe intervention that provides effective symptom control. High-volume centers report favorable long-term outcomes in 78–90% of cases [1–3]. However, 10–20% of patients experience anatomical failure over time, and 3–7% of these cases ultimately require revisional antireflux surgery [1, 4–10]. Revisional procedures are inherently more complex than primary surgeries and are associated with higher complication rates and lower symptom resolution [11, 12]. As such, a thorough preoperative evaluation is critical for optimizing patient selection and surgical planning.

Esophageal pH monitoring is an essential test in the preoperative assessment of patients undergoing primary antireflux surgery, with studies identifying an abnormal DeMeester score as the strongest predictor of favorable outcome [13, 14]. While pH monitoring is commonly performed prior to revisional ARS, its role in guiding surgical decisions and predicting outcomes remains uncertain [15]. This is largely due to the paucity of data on this topic and the diverse clinical presentations of patients with failed fundoplication.

There is a need to better understand the utility of pH monitoring in the work-up of patients with anatomical failure after ARS. This study aims to evaluate the diagnostic and prognostic value of esophageal pH monitoring in such patients. The primary objective is to assess its role in selecting candidates for revisional ARS, while the secondary objective is to evaluate its predictive value for surgical outcomes.

## Materials and methods

### Study design and population

This retrospective study analyzed prospectively collected data from patients who underwent antireflux surgery (ARS) at Allegheny Health Network hospitals (Pittsburgh, PA) between January 2015 and December 2023. Inclusion criteria were adult patients (≥ 18 years) with a diagnosis of anatomical failure following a primary Nissen or partial fundoplication. For those underwent revisional surgery a minimum of one-year follow-up after the revisional surgery was required. Only patients who underwent 48-h Bravo pH monitoring at the time of anatomical failure were selected. Patients undergoing other types of antireflux procedures, including magnetic sphincter augmentation, revisional bariatric surgery, gastric or esophageal resections, or transoral incisionless fundoplication, were excluded. Institutional Review Board (IRB) approval was obtained for this study (IRB No. 2020-0687).

### Preoperative assessment

Preoperative workup included a detailed clinical history, barium contrast study, upper gastrointestinal endoscopy, esophageal manometry, and 48-h pH monitoring. All patients completed a comprehensive foregut evaluation, including the GERD-Health-Related Quality of Life (GERD-HRQL) questionnaire before revisional ARS [16]. This 16-item disease-specific tool evaluates symptoms such as heartburn, regurgitation, dysphagia, and gas bloating. Patients also reported their overall satisfaction and proton pump inhibitor (PPI) use.

### Upper gastrointestinal endoscopy

Endoscopy, performed by experienced foregut surgeons, assessed key anatomical features, including crural impression, gastroesophageal junction (GEJ) position, fundoplication integrity, and the presence of esophagitis graded by Los Angeles classification. Anatomical failure was defined as recurrent hiatal hernia, disruption, or slippage of the fundoplication [17, 18].

- *Recurrent hiatal hernia* was identified if the GEJ was above the crura, with hernia size measured as the distance from the GEJ to the crural level.
- *Fundoplication disruption* was categorized as total (absence of visible folds) or partial (loose or distorted folds).
- *Slipped fundoplication* was diagnosed when the wrap encircled the stomach instead of the distal esophagus, with a secondary narrowing observed below the GEJ.

### Esophageal pH monitoring

All patients underwent 48-h Bravo (Medtronic, Shoreview, MN) pH monitoring at the time of the anatomical failure was determined. Proton pump inhibitors were discontinued for ten days prior to testing. Distal esophageal acid exposure was considered abnormal with a DeMeester score > 14.7, consistent with prior literature [19].

### Videoesophagram

Videoesophagrams assessed gross pharyngeal and esophageal motility and identified anatomical abnormalities, including masses, mucosal lesions, diverticula, strictures, or hiatal hernias.

### High-resolution impedance manometry (HRIM)

High-resolution impedance manometry was performed using a 4.2-mm solid-state catheter with 36 pressure sensors (Medtronic, Minneapolis, MN). Sensors were calibrated and then a baseline measurement was taken before the measurement of ten serial liquid swallows 20 s apart. Manometric variables assessed included lower esophageal sphincter characteristics, esophageal motility, and bolus clearance, analyzed with Manoview software. (Medtronic, Minneapolis, MN).

### Surgical procedure

All revisional ARS procedures were performed laparoscopically. The procedure initiated with meticulous lysis of adhesions to provide better exposure of the hiatal region and visualize the previous fundoplication. After identification of the crural pillars, the distal esophagus was circumferentially mobilized, and the herniated and/or disrupted fundoplication was carefully reduced and taken down. Restoration of the > 3 cm intra-abdominal esophageal length was achieved and new fundoplication was reconstructed. The hiatal opening was repaired without the use of pledgets, biologic mesh was used selectively. Particular attention was given to the identification and preservation of the anterior and posterior vagal nerve trunks throughout the procedure. Intraoperative endoscopy was performed to confirm the integrity of the reconstructed fundoplication and assess luminal resistance at the distal esophagus. All procedures were performed by a consistent group of high-volume foregut surgeons adhering to standardized operative and postoperative management protocols, which helped ensure procedural uniformity and reduce variability over the study period.

### Postoperative outcome assessment

Postoperative evaluations were conducted at 2 weeks, 6 weeks, 6 months, and annually after primary and revisional antireflux surgery. During these visits, patients were assessed for disease-specific symptoms, use of antisecretory medications, and procedure-related complications. In addition, patients were asked to complete the GERD-HRQL questionary at their 1-year follow-up. Our primary outcome was defined using a composite criterion of both patient satisfaction and freedom from proton pump inhibitor use to ensure clinical relevance and minimize false-positive results from single endpoints. Additionally, the Bonferroni method was used for adjusting p values regarding secondary outcome comparison for GERD-HRQL total score.

### Statistical analysis

Patients were stratified into groups based on normal or abnormal DeMeester scores at the time of anatomical failure. Demographics, clinical characteristics, and endoscopic findings were compared to address the study’s primary goal of assessing pH monitoring’s role in guiding surgical decisions. Additionally, favorable outcome at one-year post revisional antireflux surgery were analyzed with respect to the DeMeester score, along with all other clinical factors to address secondary goal of outcome prediction. Finally, impact of DeMeester score at the time of failure on outcome of revisional ARS was assessed in subgroups based on the primary symptom at failure (reflux, dysphagia), the timing of failure (< 6 months, > 5 years), and the pattern of failure (herniated, disrupted, herniated and disrupted).

Data were expressed as median (interquartile range) or mean (standard deviation) for continuous variables, frequency and percentage for categorical variables. The Fisher exact test and the Mann–Whitney *U* test were conducted where appropriate. Univariate logistic regression analysis was utilized to identify potential predictors for an outcome, then a multivariable logistic regression analysis selection procedure was performed, where a predictor with significant entry level 0.3 and significant stay level 0.1 was selected into the model. To account for potential temporal variation over the study period, both multivariable logistic regression models were repeated with adjustment for year of initial surgery. These year-adjusted models were used to evaluate whether evolving surgical or postoperative practices influenced the identified predictors of need for revisional surgery and favorable outcomes. Additionally, sensitivity analysis with multiple imputation methodology under the missing at random (MAR) assumption was conducted after a multivariable model was fitted. Statistical significance was defined as a *p*-value < 0.05 for all analyses. All statistical analyses were performed using Statistical Analysis System (SAS) software (version 9.4, SAS Institute, Cary, NC).

## Results

### Study population

The study population consisted of 278 patients who were diagnosed with anatomical failure following primary Nissen or partial fundoplication. All patients underwent 48-h pH monitoring at the time of failure. The median time to diagnosis of anatomical failure from the initial ARS was 37.5 months (IQR 15.5–100.6). Demographic characteristics, clinical data, and failure patterns for the study population are summarized in Table 1.

**Table 1:** Demographic characteristics, clinical data, and failure patterns of study population

Characteristics	Total patients (*N* = 278)
Age, y, median (IQR)	57 (47–66)
Sex (Female), N (%)	190 (68.3%)
BMI, median (IQR)	28.4 (25.5–32.7)
BMI ≥ 30, N (%)	112 (40.3%)
Anatomical failure type, N (%)	
Herniated fundoplication only	108 (38.8%)
Both herniated and disrupted	99 (35.6%)
Slipped/disrupted fundoplication only	71 (25.5%)
GERD-HRQL score, median (IQR)	
Total score	20.0 (6.0–37.0)
Heartburn score	9.0 (0.0–16.0)
Regurgitation score	6.0 (0.0–13.0)
Dysphagia score	2.0 (0.0–3.0)
PPI-use, N (%)	128 (54.0%)
Patient satisfaction, N (%)	102 (43.0%)
Esophagitis, N (%)	77 (27.7%)
Esophagitis LA C or D, N (%)	21 (7.6%)
Revisional antireflux surgery, N (%)	160 (57.6%)
Time to anatomical failure (month), median (IQR)	37.5 (15.5–100.6)

### Comparison of abnormal vs normal DeMeester score at time of failure

An abnormal DeMeester score was identified in 132 patients (47.5%) at the time of failure. Demographics and outcomes at time of failure are compared between DeMeester score status groups in Table 2. Patients with abnormal DeMeester scores were more likely to have both herniated and disrupted fundoplication (47.1 vs. 24.0%, *p* < 0.001). They had a higher GERD-HRQL heartburn score (*p* = 0.022). Their time to diagnosis of anatomical failure was significantly longer (*p* = 0.004). They were also more likely to undergo revisional ARS than patients with a normal DeMeester score at time of failure (*p* < 0.001).

**Table 2:** Comparison of demographic and clinical characteristics at the time of the anatomical failure between the patients based on DeMeester score status at time of failure

Characteristics	Normal DeMeester score (*N* = 146)	Abnormal DeMeester score (*N* = 132)	*p* value
Age, y, median (IQR)	59 (48–67)	55 (46–64)	0.203
Sex (Female), N (%)	103 (70.5%)	87 (65.9%)	0.440
BMI, median (IQR)	28.2 (25.2–32.0)	28.7 (26.1–33.1)	0.302
BMI ≥ 30, N (%)	54 (37.0%)	58 (43.9%)	0.271
Anatomical failure type, N (%)			
Herniated fundoplication only	62 (42.5%)	46 (34.8%)	< 0.001
Both herniated and disrupted	35 (24.0%)	64 (48.5%)	
Slipped/disrupted fundoplication only	49 (33.6%)	22 (16.6%)	
GERD-HRQL score, median (IQR)			
Total score	17.0 (5.0–33.0)	22.5 (6.0–39.0)	0.093
Heartburn score	5.0 (0.0–14.0)	10.5 (1.0–18.0)	0.022
Regurgitation score	5.0 (0.0–12.0)	7.5 (0.0–15.0)	0.070
Dysphagia score	2.0 (0.0–3.0)	2.0 (0.0–4.0)	0.548
PPI-use, N (%)	56 (45.5%)	72 (63.2%)	0.009
Patient satisfaction, N (%)	56 (45.5%)	46 (40.4%)	0.434
Esophagitis, N (%)	30 (20.5%)	47 (35.6%)	0.007
Esophagitis LA C or D, N (%)	3 (2.1%)	18 (13.6%)	< 0.001
Revisional antireflux surgery, N(%)	69 (47.3%)	91 (68.9%)	< 0.001
Time to anatomical failure (month), median (IQR)	27.9 (14.8–77.8)	54.9 (20.9–121.0)	0.004

### Predictors of the need for revisional antireflux surgery

There were 160 patients with anatomical failure (57.6%) who required revisional surgery at a median of 68.6 months (IQR 21.1–181.5) after their index operation, 151 patients (94.4%) underwent revision to a Nissen fundoplication and 9 patients (5.6%) underwent revision to a partial fundoplication.

Demographic, clinical, and objective testing factors at the time of failure are compared between patients who did and did not require revisional ARS in Table 3. Patients who underwent revisional surgery were more likely to be dissatisfied with the outcome of their index surgery (78.3 vs. 27.3%, *p* < 0.001) and to be taking PPIs (79.7 vs. 18.2%, *p* < 0.001). They also had higher GERD-HRQL total scores, as well as higher scores for heartburn, regurgitation, dysphagia, and gas-bloating. Notably, patients who underwent revision were more likely to have an abnormal DeMeester score (56.9 vs 34.7%, *p* < 0.001).

**Table 3:** Univariate logistic regression analysis of potential demographic and outcome at the time of failure predictors of need for revisional antireflux surgery

Characteristics	No Revisional ARS (*N* = 118)	Revisional ARS (*N* = 160)	Odds ratio (95% CI)	*p* value
Age, y, median (IQR)	62 (52–69)	54 (43–63)	0.95 (0.93–0.97)	< 0.001
Age ≥ 60, N (%)	73 (61.9%)	52 (32.5%)	0.30 (0.18–0.49)	< 0.001
Sex (Female), N (%)	79 (66.9%)	111 (69.6%)	1.12 (0.67–1.86)	0.665
BMI, median (IQR)	28.3 (24.9–33.5)	28.4 (26.1–32.6)	1.00 (0.95–1.05)	0.845
BMI ≥ 30, N (%)	50 (42.4%)	62 (38.8%)	0.86 (0.53–1.39)	0.544
Anatomical failure type, N (%)				
Herniated fundoplication only*	59 (50.0%)	49 (30.6%)		
Both herniated and disrupted	21 (17.8%)	78 (48.8%)	4.39 (2.38–8.09)	< .001
Disrupted fundoplication only	30 (25.4%)	27 (16.9%)	1.08 (0.57–2.06)	0.806
Slipped fundoplication only	8 (6.8%)	6 (3.8%)	0.92 (0.30–2.83)	0.883
GERD-HRQL score, median (IQR)				
Total score	7.0 (3.0–19.0)	32.0 (17.0–49.0)	1.07 (1.05–1.09)	< 0.001
Heartburn score	0.9 (0.0–7.0)	13.0 (6.0–20.0)	1.16 (1.11–1.21)	< 0.001
Regurgitation score	0.0 (0.0–6.0)	12.0 (3.0–18.0)	1.16 (1.11–1.22)	< 0.001
Dysphagia score	1.0 (0.0–2.0)	3.0 (1.0–4.0)	1.49 (1.26–1.76)	< 0.001
GERD-HRQL total score (≥ 15) N (%)	31 (31.3%)	108 (78.3%)	7.74 (4.31–13.89)	< 0.001
PPI-use, N (%)	18 (18.2%)	110 (79.7%)	17.08 (8.88–32.85)	< 0.001
Patient satisfaction, N (%)	72 (72.7%)	30 (21.7%)	0.11 (0.06–0.19)	< 0.001
Esophagitis, N (%)	26 (22.0%)	51 (31.9%)	1.64 (0.95–2.84)	0.076
Esophagitis LA C or D, N (%)	7 (5.9%)	14 (8.8%)	1.47 (0.58–3.75)	0.419
DeMeester score; median (IQR)	6.8 (1.9–22.3)	20.4 (5.4–52.5)	1.02 (1.01–1.03)	< 0.001
Abnormal DeMeester score, N (%)	41 (34.7%)	91 (56.9%)	2.46 (1.51–4.02)	< 0.001
Time to anatomical failure (month), median (IQR)	22.8 (12.6–48.3)	68.6 (21.1–181.5)	1.02 (1.01–1.02)	< 0.001

*Odd’s ratios for anatomical failure type were calculated in reference to herniated fundoplication

Significant and borderline predictors identified on univariate analysis were included in a multivariable logistic regression model. The three independent predictors for the need for revisional surgery were: GERD-HRQL total score > 15, abnormal DeMeester score, and age < 60 years (Table 4). Of note, GERD-HRQL total score > 15 was the strongest predictor of need for revisional ARS [OR:7.38 (4.00–13.65), *p* < 0.001].

**Table 4:** Independent predictors of need for revisional antireflux surgery on multivariable analysis

Predictor	Estimate (SE)	Odds ratio (95% CI)	*p* value
Abnormal DeMeester score	0.86 (0.31)	2.36 (1.28–4.37)	0.006
GERD health-related quality-of-life score ≥ 15	2.00 (0.32)	7.38 (4.00–13.65)	< 0.001
Age < 60	0.98 (0.31)	2.66 (1.44–4.90)	0.002

### Impact of DeMeester score at time of failure on outcome of revisional ARS

Among the 160 patients who underwent revisional ARS, 91 (56.9%) had an abnormal DeMeester score at the time of failure. Clinical outcomes at a mean (SD) follow-up of 14 (3) months after revisional ARS are compared between patients who had a normal vs abnormal DeMeester score at time of failure in Table 5. Patients with abnormal DeMeester scores had lower GERD-HRQL total scores, higher satisfaction rates, and higher rate of freedom from PPI use after revisional surgery. Favorable outcome, defined as both patient satisfaction and freedom from PPI use, was achieved in 71.1% of patients with abnormal DeMeester scores, compared to 48.3% of those with normal scores (*p* = 0.007).

**Table 5:** Comparison of outcomes at 1-year follow-up after revisional antireflux surgery based on DeMeester score status at time of failure

Characteristics	Normal DeMeester score (*N* = 69)	Abnormal DeMeester score (*N* = 91)	*p* value
GERD-HRQL score, median (IQR)			
Total score	14.0 (6.0–32.0)	7.0 (2.0–21.5)	0.003
Heartburn score	4.0 (0.0–13.0)	1.0 (0.0–7.0)	0.056
Regurgitation score	2.0 (0.0–12.0)	0.0 (0.0–7.5)	0.094
Dysphagia score	2.0 (0.0–3.0)	0.0 (0.0–2.0)	0.014
PPI-use, N (%)	23 (39.7%)	17 (22.4%)	0.037
Patient satisfaction, N (%)	38 (65.5%)	63 (82.9%)	0.026

### Predictors of favorable outcomes after revisional antireflux surgery

Demographic, clinical, and objective testing factors at the time of failure are compared between patients who did and did not achieve favorable outcome after revisional ARS in Table 6. Patients who achieved favorable outcome had higher GERD-HRQL total scores and higher scores for heartburn, regurgitation, and dysphagia at the time of anatomical failure. Additionally, patients with favorable outcomes were more likely to have an abnormal DeMeester score (65.9 vs. 42.3%, *p* = 0.011) at time of failure.

**Table 6:** Univariate logistic regression analysis of potential demographic and outcome at the time of failure predictors of favorable outcomes after revisional antireflux surgery

Characteristics	Favorable outcome (*N* = 82)	Unfavorable outcome (*N* = 52)	Odds ratio (95% CI)	*p* value
Age, y, median (IQR)	54 (47–63)	52 (42–63)	1.00 (0.98–1.03)	0.846
Age > 60, N (%)	26 (31.7%)	18 (34.6%)	0.88 (.42–1.83)	0.722
Sex (Female), N (%)	54 (65.9%)	37 (71.2%)	0.79 (0.37–1.68)	0.540
BMI, median (IQR)	28.1 (26.0–32.0)	29.7 (27.2–33.3)	0.94 (0.87–1.02)	0.114
BMI > 30, N (%)	31 (37.8%)	25 (48.1%)	0.66 (0.33–1.33)	0.246
Anatomical failure type, N (%)				
Herniated fundoplication only*	24 (29.3%)	17 (32.7%)		
Both herniated and disrupted	45 (54.9%)	19 (36.5%)	1.67 (0.74–3.78)	0.222
Disrupted fundoplication only	10 (12.2%)	13 (25.0%)	0.56 (0.20–1.56)	0.264
Slipped fundoplication only	3 (3.7%)	3 (5.8%)	0.72 (0.13–3.98)	0.701
GERD-HRQL score, median (IQR)				
Total score	26.0 (12.5–39.5)	39.0 (6.0–59.0)	0.97 (0.95–0.99)	0.002
Heartburn score	12.0 (4.0–17.5)	18.0 (10.0–23.0)	0.95 (0.91, 0.99)	0.017
Regurgitation score	10.0 (1.0–14.5)	15.0 (7.0, -22.0)	0.95 ( 0.91, 0.99)	0.010
Dysphagia score	2.0 (0.0–3.0)	3.0 (2.0–4.0)	0.69 ( 0.55–0.88)	0.002
PPI-use, N (%)	54 (75.0%)	41 (87.2%)	0.46 (0.17–1.25)	0.129
Patient satisfaction, N (%)	19 (26.4)	8 (17.0%)	1.69 (0.68–4.25)	0.261
Esophagitis, N (%)	27 (32.9%)	15 (28.8%)	1.20 (0.56–2.55)	0.638
Esophagitis LA C or D, N (%)	8 (9.8%)	1 (1.9%)	3.92 (0.61–25.27)	0.151
DeMeester score, median (IQR)	29.6 (9.2–64.1)	13.1 (4.1–48.2)	1.02 (1.00–1.03)	0.011
Abnormal DeMeester score, N (%)	54 (65.9%)	22 (42.3%)	2.59 (1.27–5.29)	0.009
Time to anatomical failure (month), median (IQR)	88.8 (24.1–182.3)	45.7 (18.3–128.8)	1.00 (1.009–1.01)	0.129

*Odd’s ratios for anatomical failure type were calculated in reference to herniated fundoplication

On multivariable analysis, an abnormal DeMeester score was the strongest independent predictor of favorable outcomes, followed by the absence of dysphagia (Table 7).

**Table 7:** Independent predictors of favorable outcomes after revisional antireflux surgery on multivariable analysis

Predictor	Estimate (SE)	Odds ratio (95% CI)	*p* value
Abnormal DeMeester score	1.38 (0.42)	3.98 (1.75–9.04)	0.001
Absence of dysphagia	1.25 (0.44)	3.49 (1.47–8.29)	0.005

### DeMeester score as a predictor of outcomes of revisional ARS subgroups

**Fig. 1:**
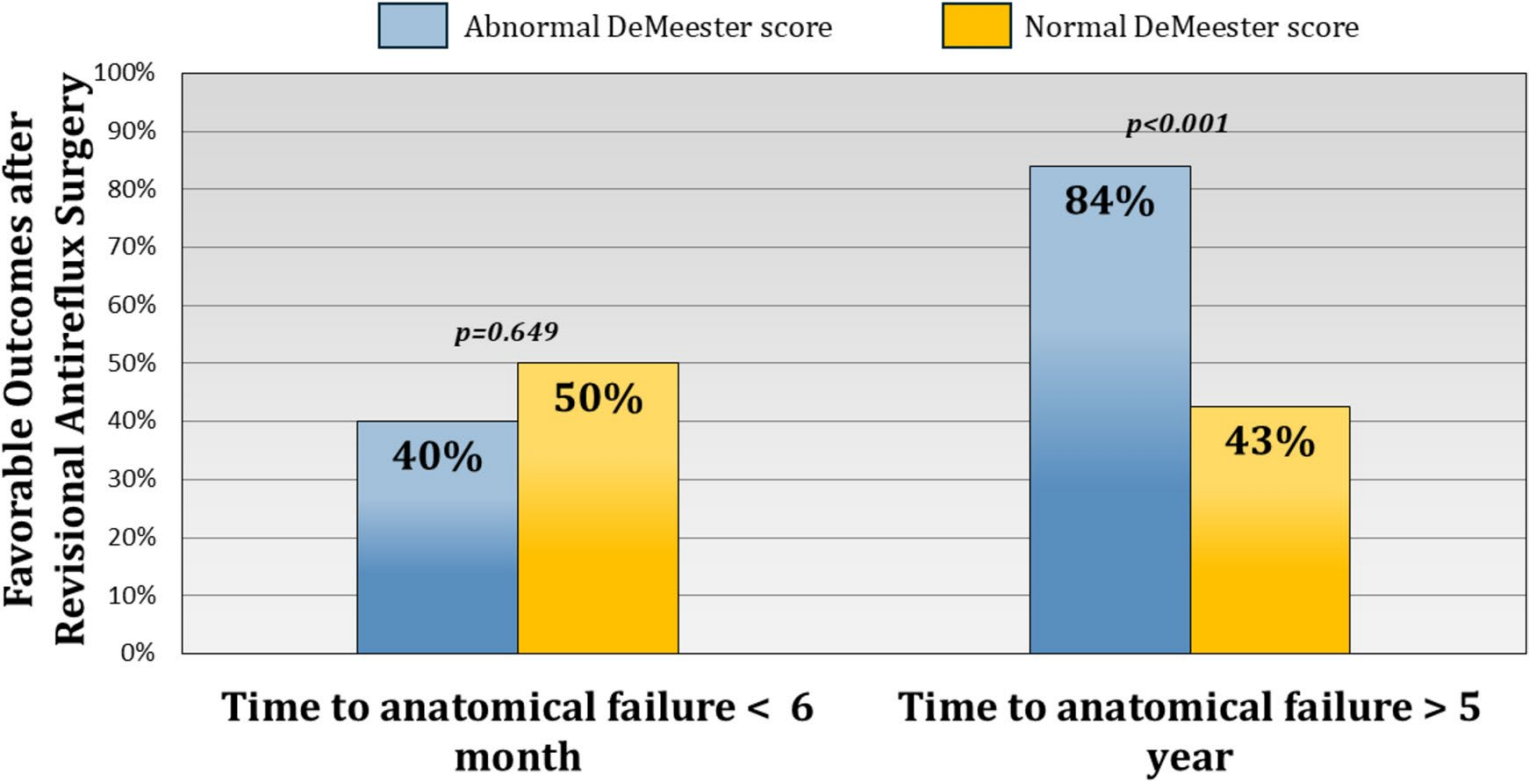
Impact of abnormal DeMeester score on revisional outcomes by failure timing. In patients with early failure (≤ 6 months post-index operation), abnormal DeMeester score was not predictive of outcome (*p* = 0.649). However, a favorable outcome was observed in late failures (≥ 5 years, 84.0 vs. 43.0%, *p* < 0.001)

**Fig. 2:**
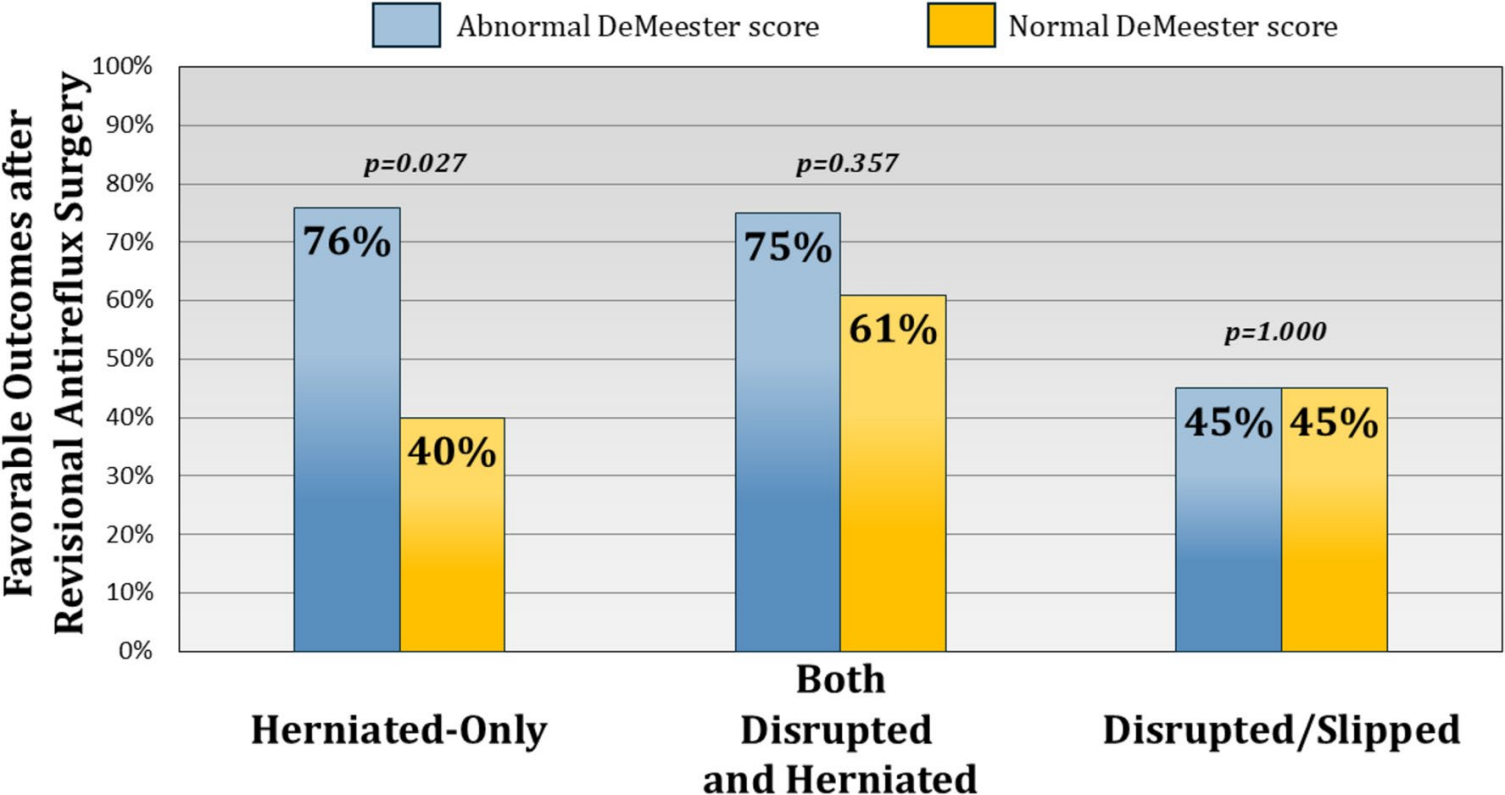
Impact of abnormal DeMeester score on revisional surgery outcomes based on failure pattern. It was associated with better outcomes in herniated-type failure (76.2 vs. 40.0%, *p* = 0.027). No significant association was observed in patients with combined disrupted and herniated fundoplication (*p* = 0.357) or those with only disrupted/slipped fundoplication (*p* = 1.000)

Subgroup analysis revealed that an abnormal DeMeester score was associated with favorable outcomes in patients presenting with recurrent reflux symptoms (71.0 vs. 47.1%, *p* = 0.018). In contrast, pH monitoring had no impact on outcomes in patients with dysphagia (*p* = 0.612). In patients with early failure (within 6 months of index operation), abnormal DeMeester score was not associated with outcome (*p* = 0.649) (Fig. 1). There was a relationship with later incidence of failure. When failure occurred five or more years after the index operation DeMeester score was associated with favorable revisional outcome (84.0 vs 43.0%, *p* < 0.001). Among failure patterns, abnormal DeMeester score was associated with favorable outcomes only in the herniated type (76.2 vs. 40.0%, *p* = 0.027). It had no significant impact on outcomes in patients with combined disrupted and herniated fundoplication (*p* = 0.357) or those with only disrupted/slipped fundoplication (*p* = 1.000) (Fig. 2). Additionally, among patients who underwent revisional antireflux surgery, a small subset received partial fundoplication. We performed a subgroup analysis comparing outcomes between patients who underwent revisional Nissen versus partial fundoplication and found no significant differences in favorable outcomes.

## Discussion

The assessment of post-fundoplication failure remains a clinical challenge, with no universally accepted framework for determining which patients require revisional surgery and which are most likely to achieve durable symptom resolution after revision. While pH monitoring is a cornerstone of preoperative evaluation in primary antireflux surgery (ARS) and has been identified as the strongest predictor of favorable outcomes following primary fundoplication, its role in patients with anatomical failure following fundoplication has remained poorly defined [13, 19]. There is no consensus about the role of pH monitoring in the assessment of postoperative symptoms. However, many clinicians believe that reflux symptoms alone are an unreliable marker of recurrent abnormal acid exposure [17, 20]. A prospective study including 124 patients with recurrent symptoms after fundoplication found that only 39% had an abnormal DeMeester score 17 months after surgery [21]. However, in a symptomatic patient with a confirmed disrupted, herniated or slipped fundoplication, the role of quantifying the degree of acid exposure often becomes secondary. This approach is supported by studies showing a strong association between anatomical failure and abnormal acid exposure, with abnormal acid exposure being found in 92% of patients with anatomical failure, but only in 10% of patients with an intact fundoplication (*p* = 0.001) [22]. In the present study we evaluated patients with anatomic failure and found that pH monitoring is not only valuable but essential in the evaluation of these patients. Abnormal acid exposure was not only a primary determinant of surgical selection but also emerged as the strongest independent predictor of postoperative success, offering critical prognostic value. To further validate this finding, we adjusted our multivariable models by year of initial surgery, and abnormal DeMeester score remained a significant independent predictor for both the need for revisional surgery and favorable postoperative outcomes. These results support the robustness and long-term prognostic relevance of abnormal acid exposure in this surgical population. In addition, sensitivity analyses using multiple imputation under the missing-at-random (MAR) assumption confirmed the stability of our findings, with only minor changes in estimates and standard errors. Patients with abnormal acid exposure experienced higher rates of symptom resolution and proton pump inhibitor (PPI) discontinuation, independent of symptom severity or anatomical findings. These results suggest that while structural failure may often suggest abnormal acid exposure, pH monitoring is required to identify those most likely to benefit from intervention. Given that the goal of revisional ARS is not merely to correct anatomical defects but to restore physiological competence at the gastroesophageal junction, these findings support the systematic incorporation of pH monitoring as an essential component of pre-revisional assessment. This approach ensures that surgical intervention is targeted toward patients who are most likely to achieve sustained symptomatic relief.

Prior studies focused primarily on the correlation between anatomical failure and pH monitoring, but they did not assess the prognostic value of pH monitoring for revisional outcomes [22–24]. A multicenter study of 309 patients undergoing primary fundoplication found that abnormal postoperative acid exposure was associated with higher rates of surgical reintervention (18 vs. 4%, *p* = 0.001) [25]. Similarly, a longitudinal analysis of 127 patients reported that the prevalence of anatomical failure increased from 15% at 3–5 years to 31% at 7–9 years postoperatively. This was paralleled by a significant rise in the prevalence of abnormal acid exposure (from 17 to 50%) and esophagitis (from 8 to 26%) [26]. However, these studies primarily assessed failure rates over time rather than the clinical implications of pH monitoring for revisional surgery selection. Other studies have focused on the disconnect between symptoms and objective reflux burden, rather than prognostic utility. A retrospective review of 228 patients undergoing pH monitoring after fundoplication found that while recurrent heartburn had a positive predictive value of just 42% for detecting abnormal acid exposure, anatomical failure was strongly associated with pH abnormalities [23]. Additionally, a single-center study of 86 patients undergoing postoperative pH monitoring found that those with anatomical failure had a 52.6-fold increased risk of pathological acid exposure, with 75% demonstrating abnormal acid exposure compared to only 16% in those with intact fundoplications [24]. These findings confirm that pH monitoring is highly effective in identifying anatomical failure; however, they do not address whether it can predict which patients are likely to achieve symptomatic relief following revisional antireflux surgery (ARS). Our study shifts the focus from failure detection to outcome prediction, demonstrating that pH monitoring is not simply a diagnostic adjunct but a critical tool for pre-revisional risk stratification. While patient symptoms, as reflected by elevated GERD-HRQL scores, were the strongest determinant for the decision to operate, an abnormal DeMeester score was also a key factor in surgical selection. Crucially, pH monitoring has emerged as the strongest independent predictor of surgical success. Notably, even after applying Bonferroni correction for multiple secondary outcomes, the improvement in GERD-HRQL total scores remained statistically significant (adjusted *p* = 0.018), further supporting the clinical relevance of this composite symptom measure. This distinction holds clinical significance: structural failure alone does not necessarily guarantee symptom resolution after revision, whereas pH monitoring helps identify patients most likely to experience sustained relief following surgery. Given that prior research has largely examined pH monitoring as a diagnostic tool rather than a prognostic marker, our findings underscore its essential role in refining patient selection and optimizing surgical outcomes.

Our study identified pH monitoring as the strongest independent predictor of favorable revisional outcomes; however, its prognostic value was not uniform across all patient subgroups. Among patients undergoing revision for dysphagia or early failure (≤ 6 months after primary surgery), pH monitoring did not distinguish between those who achieved symptom relief and those who did not. These findings suggest that symptom recurrence in these populations arises from mechanisms unrelated to pathological reflux, such as mechanical obstruction or esophageal motility dysfunction [27, 28]. Prior studies reinforce this concept. A retrospective analysis of 144 revisional surgeries found that patients undergoing revision for dysphagia were significantly more likely to have an intact fundoplication compared to those presenting with recurrent reflux (33.3 vs. 7.7%, *p* = 0.01), while patients with heartburn exhibited higher rates of wrap disruption (43.6 vs. 18.5%, *p* = 0.03) [29]. Similarly, a systematic review of 4584 revisional ARS cases reported that patients undergoing revision for dysphagia had significantly lower total acid exposure times compared to those with recurrent reflux (1.4 vs. 4.8%, *p* < 0.05) [12]. This aligns with findings from a postoperative pH monitoring study, which found that dysphagia was not associated with abnormal esophageal acid exposure, further supporting the notion that non-reflux mechanisms contribute to persistent symptoms in this subgroup [24]. Early failure follows a different pattern. Rather than a gradual decline in reflux control, failure occurring within six months of primary fundoplication is often linked to technical factors such as technical errors in fundoplication construction or excessive tension on the repair [30–33]. As a result, pH monitoring does not reliably predict outcomes in these cases, since the predominant failure mechanism is mechanical rather than physiological [20]. These findings reinforce the heterogeneity of post-fundoplication failure and highlight the importance of tailoring pre-revisional assessment based on failure phenotype rather than relying solely on pH monitoring.

Older patients were significantly less likely to undergo revisional antireflux surgery (ARS), despite age not being an independent predictor of postoperative success. This trend is consistent with prior large-scale analyses, including a New York statewide study of 9,462 fundoplications, which found that patients aged 30–49 years had an odds ratio of 2.01 (95% CI 1.31–3.14) for requiring revision compared to those aged ≥ 70 years, while patients aged 50–69 years had an odds ratio of 1.61 (95% CI 1.08–2.44) [6]. Similarly, a California statewide database study reported significantly higher rates of reoperation in patients aged 30–50 years (HR = 1.89) and 50–65 years (HR = 1.65) compared to those aged ≥ 65 years [34]. These studies suggest that younger patients may be more likely to undergo revision due to longer life expectancy, greater ability to tolerate reoperative procedures, and possibly a lower threshold for surgical intervention. Despite these differences in surgical selection, our study found that age did not influence postoperative GERD-HRQL scores, symptom resolution, or freedom from proton pump inhibitors (PPIs), indicating that older patients derive comparable benefits from revisional ARS when appropriately selected. This concept is consistent with a study of 492 patients who underwent ARS and found that advanced age was associated with more complications (OR 2.9, *p* = 0.003) but comparable quality of life outcomes [35]. These findings reinforce that age alone should not dissuade patients from considering revision, particularly when symptoms significantly impact quality of life. While comorbidities and surgical risk must be evaluated on an individual basis, our results suggest that revisional surgery should remain a viable option for symptomatic older patients with anatomical failure.

This study evaluated the role of pH monitoring in the assessment of patients with anatomical failure of fundoplication for revisional surgery, specifically those with herniation, slippage, or disruption. By restricting the cohort to patients with structural failure and abnormal acid exposure, this analysis provides a focused assessment of reflux burden in those most likely to undergo reoperation based on anatomical disruption and symptom severity. However, the role of pH monitoring in evaluating patients with symptoms despite an intact fundoplication remains unknown. While it may have clinical value in that setting, our findings should not be extrapolated to guide surgical decision-making in the absence of anatomical failure. These results highlight the need to determine whether abnormal acid exposure predicts revisional outcomes in anatomically intact patients or if alternative mechanisms, such as esophageal hypersensitivity or motility disorders, drive symptoms in that group.

This retrospective study is inherently subject to limitations in controlling for potential confounders, such as variations in surgical technique and patient adherence. Nevertheless, all procedures were performed by a small, consistent team of high-volume foregut surgeons using standardized operative and postoperative protocols, which minimizes these sources of variability. Although the study spanned a long period, our multivariable models were adjusted for year of surgery, and no significant impact was observed on the primary outcome predictors. The median follow-up of 14 months following revisional surgery provides medium-term outcome data regarding favorable clinical response, though longer follow-up is necessary to evaluate anatomical recurrence. Lastly, while additional pH metrics such as acid exposure time (AET) and symptom association probability (SAP) were not included, prior literature and internal analysis demonstrated strong correlation between AET and DeMeester score [13, 36]. Furthermore, our previous work has shown that SAP does not independently affect surgical outcomes in the setting of abnormal acid exposure [37].

## Conclusion

This study evaluated the role of pH monitoring in patients with anatomical failure following primary Nissen or partial fundoplication. We analyzed its ability to predict the need for revisional surgery and postoperative outcomes. Patients with abnormal acid exposure were more than twice as likely to require reoperation and nearly four times as likely to experience postoperative symptom resolution. These findings suggest that pH monitoring is most valuable for risk stratification rather than failure detection. Several factors influenced the need for revisional surgery: recurrent GERD symptoms, anatomical failure confirmed by endoscopy and pH monitoring, and recurrent hernias greater than 2 cm were all associated with the need for reoperation. Younger patients were more likely to undergo revision, which may reflect differences in surgical candidacy, symptom tolerance, or selection bias in referral patterns. Abnormal acid exposure did not predict revisional outcome in all patients. In patients undergoing revision for dysphagia or those early failure pH monitoring had limited prognostic value. In these subgroups, mechanical dysfunction rather than acid exposure was likely the primary driver of symptoms. By clarifying the role of pH monitoring in risk stratification, this study refines pre-revisional patient selection. It ensures that surgical intervention is directed toward patients most likely to achieve durable symptomatic relief. These findings establish pH monitoring as an essential tool in revisional surgery, reinforcing the need for a more individualized, evidence-based approach to optimize patient outcomes.
